# Supplementary material for: Human Preferences for Dogs and Cats in China: The Current Situation and Influencing Factors of Watching Online Videos and Pet Ownership
Source: Animals (Basel). 2024 Nov 29;14(23):3458. doi: 10.3390/ani14233458 (PMC11640227; doi:10.3390/ani14233458)

Table S1: Classification of 96 popular channels into 15 types of human's preferences from the website Bilibili.

Preference types	Channels (key words)	Preference types	Channels (key words)
Pets	Cute pets	Game	DNF
	Dogs		STEAM
	Cats		Honkai Impact 3rd
Culture	Cosplay		PUBG
	Korean		Single player game
	Chinese tradition costume		Identity V
	History		Individual games
	USA		Game for Peace
	Humanity		Arknights
	Japan		Overwatch
	Voice acting		Honor of Kings
	House dancing		Minecraft
Education	Public classes		Games
	Examinations		Onmyoji
	Postgraduate entrance		Audio games
	Speaking		League of Legends
	English tests		League of Legends mobile game
	English learning		Game Highlights
Entertainment	TV shows		Genshin Impact
Fashion	Color & makeups		Board games
	Fashion trend	Music	KPOP
	Outfit		Electronic music
	Hairstyle		Cover
	Clothing		Classic music
	Skin care		European & American music
	Makeup tutorial	Science & technology	Programming
	Makeups		Computer
Food	Foodie		Science fiction
	Cooking skills		Popular science
	home cooking		Science
	Delicious food		Car
	Dessert		Cell phone
Fun	Blackpink	Shopping	Shopping sharing
	Funny videos		Online shopping
	Autotune remix		Advertisement
	Autotune training	Sport & Health	Lose weight
	Shuyi Zhou		Healthy
Handcraft & art	DIY		Fitness
	Painting		Basketball
	Tech nerd		Physical sport
	Hand work	Other	VLOG
	Wild technology association		Video editing & recreating
Movie & TV	MAD		Finance
	Hanzawa Naoki		Travel

Movie commentary
Anime Talk
Short film
Photography
Hand comics
Comics
Movie review
Film & television editing

Table S2: Questions listed in our online anonymous questionnaire

ID	Question
1	Have your paternal grandparents ever owned a dog/cat, or do they currently own one?
2	Have your maternal grandparents ever owned a dog/cat, or do they currently own one?
3	Have your parents ever owned a dog/cat, or do they currently own one?
4	Have you ever owned a dog/cat, or do you currently own one?
5	Do you plan to own a dog/cat?
6	Do you own a dog/cat for the purpose of improving physical health?
7	Do you own a dog/cat due to your religious/cultural traditions?
8	Do you own a cat/dog to gain emotional value?
9	Do you own a dog/cat for functional reasons?
10	Do you own a dog/cat to fit into social circles or because you see others owning a dog/cat?
11	Do you prefer cats or dogs?
12	Do you live with your parents?
13	Do you believe that owning a dog/cat can compensate to some extent for the emotional absence of parents?
14	Are you married?
15	Do you have a boyfriend/girlfriend?
16	Do you believe that owning a dog/cat can compensate to some extent for the emotional absence of a boyfriend/girlfriend (or the absence of a boyfriend/girlfriend)?
17	Do you have children?
18	Do you live with your children?
19	Have your children ever owned a dog/cat, or do they currently own one?
20	Do you believe that owning a dog/cat can compensate to some extent for the emotional absence of children?
21	What is your Gender?
22	What is your living area?
23	Which city do you live in?
24	How old are you?
25	How much do you earn per month?

Figure S1. Growth trends of 15 types of preferences over 13 years (2009-2021).

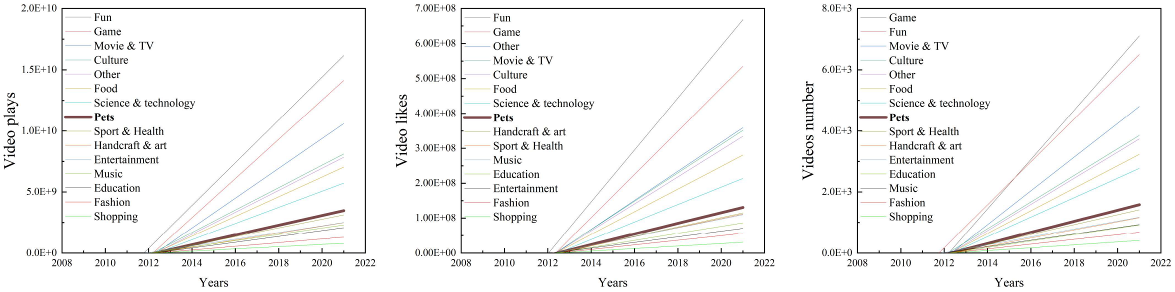

Figure S2. The proportion of respondents (yes or no) that answered a pet (or pets) could compensate the emotional void from the absence or loss of 1) parents, 2) spouse or partner, 3) children.

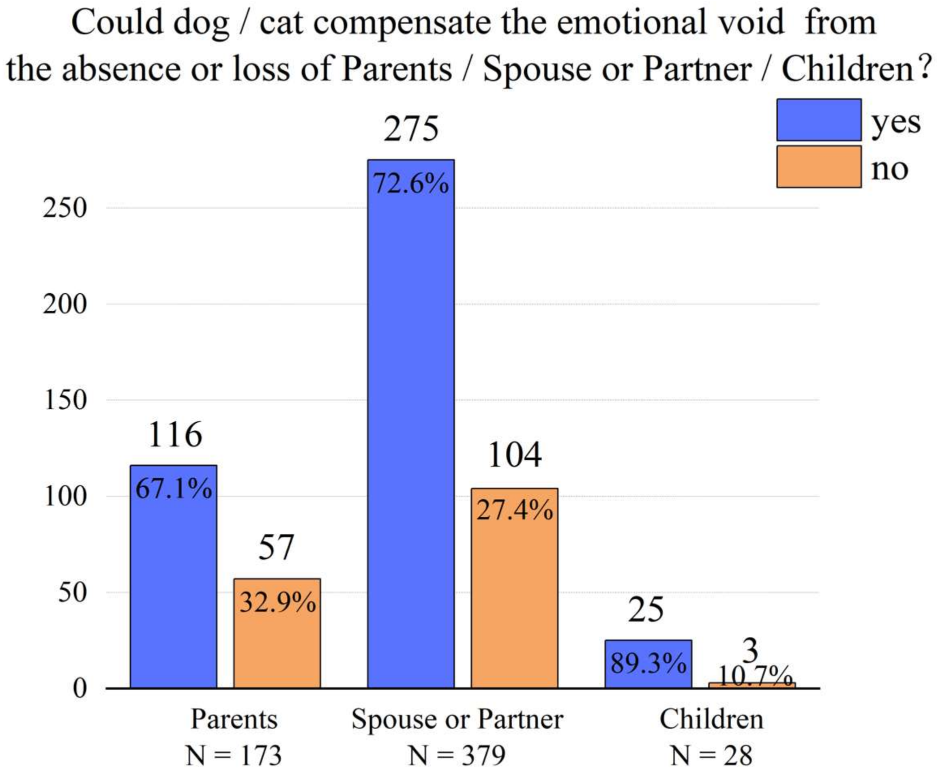

Supplement: Supplementary file 1 [file animals-14-03458-s001.zip › animals-3261832-supplementary.pdf]
